# Arginase in the Vascular Endothelium: Friend or Foe?

**DOI:** 10.3389/fimmu.2014.00589

**Published:** 2014-11-17

**Authors:** Rudolf Lucas, David Fulton, Robert William Caldwell, Maritza J. Romero

**Affiliations:** ^1^Vascular Biology Center, Medical College of Georgia, Georgia Regents University, Augusta, GA, USA; ^2^Department of Pharmacology and Toxicology, Medical College of Georgia, Georgia Regents University, Augusta, GA, USA

**Keywords:** arginase inhibitors, eNOS, reactive oxygen species, microvascular permeability, impaired vasorelaxation, vessel wall remodeling, l-citrulline

This special issue, entitled “the role of arginase in endothelial dysfunction,” assembles original contributions (1–4), as well as timely reviews (5–12) broadly related to the deleterious activities of the manganese-containing enzyme arginase in the vascular endothelium. The arginase 1 isoform is cytosolic and is mainly localized in the liver, where it performs a crucial role in eliminating nitrogen formed during amino acid and nucleotide metabolism via the urea cycle. Recent studies have demonstrated that it is also expressed in extra-hepatic tissues, including the vascular endothelium. Arginase 2 is a mitochondrial enzyme expressed in various cell types, including those in the kidneys (1) and the vasculature. In blood vessels, both isoforms likely play a role in the regulation of l-arginine homeostasis and the production of l-ornithine for subsequent polyamine and proline synthesis, which are involved in endothelial and smooth muscle cell proliferation and collagen deposition (13, 14). Polyamine and proline synthesis are crucial components of physiological and pathological vascular remodeling, a topic extensively discussed in this issue by Durante (5). Since both arginase isoforms are expressed in vascular endothelial cells, they can potentially interfere with the activity of endothelial nitric oxide synthase (eNOS), by “stealing away” the common substrate l-arginine, required to generate nitric oxide (NO) and l-citrulline. NO is a crucial mediator of endothelium-dependent vasorelaxation and restricts vascular growth and inflammation (14). Therefore, excessive activation of arginase by pathologic stimuli, including bacterial toxins [e.g., LPS and pneumolysin (6)], pro-inflammatory cytokines [e.g., TNF (15)], reactive oxygen species (ROS) (7, 11), or hyperglycemia (1, 3, 4) can potentially induce severe endothelial dysfunction. This theory is, however, complicated by observations that even during dramatically increased arginase activity, the concentrations of l-arginine in endothelial cells remain sufficiently high to conceptually support eNOS-mediated NO synthesis. As such, this has led to the suggestion that there is sub-cellular compartmentalization of l-arginine into poorly interchangeable intracellular pools, a topic discussed by Chen et al. in this issue (8). The review by Yang and Ming (7) gives an overview of the direct role of arginase in eNOS dysfunction. Indeed, a pathological increase in vascular arginase activity was shown to significantly contribute to “eNOS uncoupling,” a phenomenon observed in various vascular pathologies and in aging (16), during which the enzyme generates detrimental amounts of superoxide instead of the vasoprotective NO. Decreased NO bioavailability within the vessel wall induced by competitive utilization of l-arginine by arginase and “eNOS uncoupling” can be partially circumvented by a recently discovered alternative pathway of NO generation: the reduction of nitrate and nitrite, which is the focus of the review by Madigan and Zuckerbraun (9). Since large vessel endothelial cells are functionally and morphologically distinct from microvascular endothelial cells (17), this review highlights studies on the role of arginase in both of these cell types. Using a DOCA salt-induced mouse model in wild type, Arg1^+∕−^Arg2^−/−^ mice, Toque et al. present original data demonstrating an important role of arginase 1 in impaired vasorelaxation in the aorta and in hypertension (2). Bagi et al. present a concise review on the upregulation of arginase 1 expression and its effects on eNOS function in coronary arteries from diabetic patients (10). Addressing the microvascular compartments, Johnson et al. demonstrate that arginase is an essential mediator of skeletal muscle arteriolar endothelial dysfunction in diabetes, which may further compromise glucose utilization and facilitate the development of diabetes and hypertension (3). Acute pretreatment with l-arginine or with arginase inhibitors significantly improved endothelial function in skeletal arterioles. Kuo and Hein review recent data on the role of arginase in the generation of ROS and the subsequent vasomotor dysfunction of the coronary microcirculation upon angiotensin 2 receptor activation (11). Lucas et al. discuss recent findings suggesting a role for arginase 1 in pneumolysin-induced pulmonary capillary barrier dysfunction (6), which involves impairment of eNOS function (18). They demonstrate that arginase inhibitors significantly prevent pneumolysin-induced barrier dysfunction, at least in part by preventing the loss of expression of the adherens junction protein, VE cadherin (6). Comparing streptozotocin-treated diabetic wild type with diabetic Arg1^+∕−^Arg2^−/−^ transgenic mice, Patel et al. demonstrate an important role for arginase in the pathogenesis of diabetic retinopathy and they extend these findings *in vitro* in high glucose-treated bovine retinal endothelial cells. Their results advance arginase as a potential therapeutic target for preserving NO bioavailability, limiting oxidative stress, and preventing early signs of diabetic retinopathy (4). Although acute treatment with l-arginine was shown to significantly improve endothelial function in several experimental studies, including those by Johnson et al. in this issue (3), a recent clinical trial in patients with acute myocardial infarction demonstrated that a 6-month chronic treatment with l-arginine in addition to standard postinfarct medications did not improve clinical outcomes. Moreover, there was an increased risk of death in older patients after infarction, which promoted the early termination of the trial (19). This study clearly indicates that the long-term treatment with l-arginine is not the optimal treatment for arginase-mediated endothelial dysfunction and stresses the need for alternative strategies to inhibit arginase. Given the vital role that arginase plays in the detoxification of ammonia in the urea cycle, this suggests that chronic inhibition using specific arginase inhibitors should produce severe side effects. Surprisingly, this does not happen, possibly because the expression of arginase in the liver is much higher than in the vasculature (14). The review by Steppan et al. in this issue discusses the development of novel ABH-based arginase inhibitors, apart from the classically used boronic acid derivatives and moreover addresses potential disadvantages of developing isoform-specific inhibitors for macrophage function (12). As an alternative to l-arginine therapies, l-citrulline is a product of NO synthases that can be recycled to l-arginine. Romero et al. presents data demonstrating a potent protective action of l-citrulline in streptozotocin-induced diabetic nephropathy in mice, accompanied by lower albuminuria and kidney fibrosis (1). l-citrulline also restored the NO/ROS balance and barrier function in high glucose-treated monolayers of human glomerular endothelial cells. Intriguingly, l-citrulline promoted the sustained elevation of arginase 2 expression/activity in proximal tubule epithelium of kidneys from mice. This was mediated, at least in part, via a previously unappreciated immunomodulatory ability to significantly increase plasma levels of the anti-inflammatory cytokine IL-10 (20, 21). Given the weight of experimental evidence to date, there are little doubts that arginase plays a significant role in limiting l-arginine utilization by eNOS and compromising endothelial function. Currently, acute l-arginine and arginase inhibitors are effective in improving endothelial function. However, given the important contributions of arginase to vital metabolic functions, the long-term consequences of arginase inhibition remain uncertain. Improved selectivity could perhaps be obtained by elucidating and targeting the molecular mechanisms that lead to specific upregulation of arginases in the vascular compartment.

## Conflict of Interest Statement

The authors declare that the research was conducted in the absence of any commercial or financial relationships that could be construed as a potential conflict of interest.
